# LncRNA SNHG5 promotes the glycolysis and proliferation of breast cancer cell through regulating BACH1 via targeting miR-299

**DOI:** 10.1007/s12282-021-01281-6

**Published:** 2021-08-05

**Authors:** Shu-Lin Huang, Zhong-Cheng Huang, Chao-Jie Zhang, Jing Xie, Shan-Shan Lei, Ya-Qin Wu, Pei-Zhi Fan

**Affiliations:** 1 Department of Breast and Thyroid Surgery, Hunan Provincial People’s Hospital, The First Affiliated Hospital of Hunan Normal University, No. 61, Jiefang West Road, Changsha, 410005 Hunan Province People’s Republic of China; 2 Department of General Surgery, Hunan Provincial People’s Hospital, The First Affiliated Hospital of Hunan Normal University, Changsha, 410005 Hunan Province People’s Republic of China

**Keywords:** Breast cancer, SNHG5, BACH1, miR-299, Glycolysis

## Abstract

**Background:**

Breast cancer (BC) is one of the most common malignant tumors in women. Accumulating studies have been reported that long non-coding RNA (lncRNA) SNHG5 is highly expressed in BC. However, the specific molecular mechanism of SNHG5 in BC is unclear.

**Methods:**

Gene and protein expressions in BC cell were detected by qRT-PCR and western blotting. The proliferation and cell cycle were measured using colony formation assay and flow cytometry analysis, separately. The glucose consumption and lactate production were determined by using the glucose assay kit and lactate assay kit. A dual-luciferase reporter assay was performed to measure the interaction between miR-299 and SNHG5 or BACH1.

**Results:**

SNHG5 and BACH1 expressions were increased in BC cell while miR-299 level was decreased. SNHG5 increased BACH1 expression by directly targeting miR-299. SNHG5 silencing or miR-299 overexpression suppressed the proliferation of BC cell, arrested the cell cycle in the G1 cell phase, and decreased the glucose consumption and lactate production of BC cell. However, inhibition of miR-299 or overexpression of BACH1 could reverse the inhibitory effects of sh-SNHG5 on cell proliferation and glycolysis in BC.

**Conclusion:**

SNHG5 promoted the BC cell growth and glycolysis through up-regulating BACH1 expression via targeting miR-299. These findings may improve the diagnostic and therapeutic approaches to BC.

## Introduction

Breast cancer (BC) acts as one of the most common malignancies in women, especially in developing countries, with a 5-year survival of less than 40% [1]. In recent years, distant metastasis has become the leading cause of death in BC patients [2]. Due to the research of molecular pathogenesis remains limited and the lack of targeted drugs in BC, patients who are treated with surgery, chemotherapy, or radiation always have a poor prognosis and are prone to relapse [3, 4]. Therefore, further exploration of the molecular mechanisms of BC is necessary to improve current therapeutic strategies.

The transcription factor BTB and CNC homology 1 (BACH1), is a member of Cap’n’Collar and leucine zipper family that involves in the progression of several cancers, such as prostate cancer [5] and ovarian cancer [6]. BACH1 played a key role in the development of BC. For example, BACH1 silencing inhibited bone metastasis in BC, moreover, BACH1 and its target genes were reported to be associated with the high risk of BC recurrence in patients [7]. It was also revealed to facilitate BC metastasis by acting as a let-7-regulated transcription factor to induce matrix metalloproteinase 1 expression [8]. BACH1 showed high expression in triple-negative breast cancer (TNBC) and its gene signature was involved in poor prognosis [9]. Besides, according to Warburg Effect, cancer cells relied on glycolysis to obtain energy [10], which suggested that the metabolic switch from oxidative phosphorylation to increased glycolysis was a vital biochemical characteristic of cancer cells including BC [11]. Increasing pieces of evidence showed an overexpression of key glycolytic enzymes, such as hexokinase (HK) pyruvate kinase (PK), in BC cells, and inhibition of these key enzymes was demonstrated to be a promising anti-cancer strategy for BC [12]. Betulinic acid was reported to suppress BC cell metastasis by regulating GRP78-mediated glycolysis [13]. Moreover, miR-30a-5p was observed to inhibit BC cell growth by suppressing LDHA-mediated glycolysis [14]. These findings suggested that glycolysis played important role in BC cell growth and metastasis. Additionally, a study indicated that BACH1 bound to the glycolytic gene Hexokinase 2 (HK2) and Glyceraldehyde-3-Phosphate Dehydrogenase (GAPDH) promoters, activated their expression and stimulated glycolysis rate, thereby promoting metastasis in lung cancer [15]. However, it is not clear whether BACH1 promotes the growth of BC cells by activating glycolysis.

Long non-coding RNAs (lncRNAs) are an important subset of non-coding RNAs with more than 200 nucleotides and have been demonstrated to affect BC progression through various mechanisms [16]. LncRNA small nucleolar RNA host gene 5 (SNHG5), a member of the non-coding multiple small nucleolar RNA host gene family, was proved to play a carcinogenic role in gastric cancer [17], hepatocellular carcinoma [18], and so on. Moreover, study also showed that SNHG5 promoted BC cell proliferation both in vivo and in vitro [19]. Nevertheless, the specific molecular mechanism remains unclear.

MicroRNAs (MiRNAs) are short non-coding RNAs with a length of 22–25 nt, which have been confirmed to be involved in a variety of cellular processes such as BC proliferation and cell cycle [20]. MiR-299 showed low expression in BC and directly targeted serine/threonine kinase 39 (STK39), knockdown of STK39 suppressed epithelial–mesenchymal transition markers and matrix metalloproteinase expression, and inhibited cell migration and invasion [21]. However, the role of miR-299 in BC proliferation is unknown. Furthermore, considering each miRNA had the potential to target a large number of genes. By bioinformatics method, we predicated there were binding sites between SNHG5 and miR-299, and BACH1 could be a target gene of miR-299, and that the interaction of these three had not been reported. Therefore, we hypothesized that SNHG5 though activating BACH1 to promote BC cell growth via targeting miR-299.

In our study, we aimed to explore the molecular mechanism of SNHG5 in the regulation of BC progression, and we found that knockdown of SNHG5 inhibited cell growth and reduced glycolysis in vitro. This finding will provide a potential target for the diagnosis and therapy of BC.

## Materials and methods

### Cell culture

The cell lines MCF-10A, MCF-7, MDA-MB-231, SK-BR-3 and MDA-MB-468 were obtained from American Type Culture Collection (ATCC, VA, USA). All cells were incubated in Dulbecco’s modified Eagle’s medium (DMEM; Gibco, CA, USA) containing 10% fetal bovine serum (FBS, Invitrogen, CA, USA), 100 U/mL penicillin, and 100 mg/mL streptomycin. Cells were cultured at 37 ℃ under humidified condition with 5% CO_2_.

### Cell transfection and vector construction

The knockdown of SNHG5 and miR-299 were performed using short hairpin RNA (shRNA) and miRNA inhibitor. The miRNA mimics and overexpression vector pcDNA3.1 were used for enhancing miR-299 and BACH1 expression. Sh-SNHG5 and sh-NC, miR-299 mimics, miR-299 inhibitor and mimics/inhibitor NC were synthesized by Genepharm (Shanghai, China). The full-length BACH1 cDNA was cloned into pcDNA3.1 (Genechem) to generate the pcDNA3.1-BACH1 vector. MDA-MA-231 and SK-BR-3 with 70% confluence were transfected with above plasmids by using Lipofectamine 3000 Reagent (Thermo Fisher Scientific, Waltham, USA).

### Quantitative real-time PCR (qRT-PCR) analysis

Total RNA was isolated by TRIzol reagent (Invitrogen). Then, RNA quality was detected using NanoDro2000c (Thermo). Next, 2 μg RNA was synthetized cDNA using the RevertAid First Strand cDNA Synthesis kit (Thermo). Process of qRT-PCR was conducted on an ABI7500 Fast Real-Time PCR System (PE Applied Biosystems) based on the standard procedures of SYBR-GreenPCR kit (Takara, Japan). β-Actin and U6 were used as the internal control for SNHG5, BACH1 and miR-299. Primers used in this study were synthesized by RiboBio (Guangzhou, China). Sequences of all primers were showed as follows:

SNHG5: F: 5’-CACAGTGGAGGAGCTCTGAA-3’, R: 5’-CTCGTGGCACTAGCCAGAAA-3’;

miR-299: F: 5’-GCTGGTTTACCGTCCCAC-3’, R: 5’-GTCGTATCCAGTGCAGGGTCCGAGGTATTCGCACTGGATACGAC-3’;

BACH1: F: 5’-CCAGAACCAGGTCAAAGGAC-3’, R: 5’-CTCAGAGTCGTCTCCCAAGC-3’;

U6: F: 5’-CTCGCTTCGGCAGCACA-3’, R: 5’-AACGCTTCACGAATTTGCGT-3’;

β-actin: F: 5’-CCCTGGAGAAGAGCTACGAG-3’, R: 5’-CGTACAGGTCTTTGCGGATG-3’.

### Cell proliferation

Transfected cells were cultured in six-well plates. The medium was changed every 2 days. After 2 weeks of incubation, colonies were stained with 0.1% crystal violet for 15 min at room temperature. Then counted the number of colonies formed to evaluate cell proliferation.

### Cell cycle analysis

Treated BC cells were seeded in six-well plates at 37 °C. After 24 h incubation, cells were trypsinized and washed twice with PBS. After centrifugation, the cells were fixed in cold 70% ethanol for 2 h. Then cells were washed with PBS twice and resuspended in 300 µL PI/RNase staining buffer (BD, NJ, USA) for 30 min at room temperature in dark. Next, the cell distribution was assayed using flow cytometry (BD) and the flowcytometric data were analyzed by ModFit LT software version 3.0 (BD).

### Glucose consumption and lactate production

The glucose consumption and lactate production were assessed by glucose assay kit (Sigma, St. Louis, USA) and lactate assay kit (Biovision, CA, USA), respectively. In brief, transfected BC cells were seeded into 12-well plates (2 × 10^5^ per well) containing glucose-free culture medium for 2 h after being cultured in a complete culture medium. The culture mediums were collected as samples for measuring lactic acid production. Then cells were lysed through ultrasonication, and the supernatants were collected as samples. Glucose probe and glucose mix kit (for glucose consumption) or lactate mix and lactate substrate mix (for lactate production) were added into the samples, after 30 min of incubation at room temperature, the absorbance of each well was detected by a microplate reader (BioRed, CA, USA) at 505 nm (for glucose assay) and 530 nm (for lactate assay).

### Dual-luciferase reporter assay

The wild-type (WT) and mutant (MUT) sequences of SNHG5 or BACH1 which contained miR-299 binding sites were amplified and inserted into pGLO vector, individually. And established recombinant luciferase reporter plasmids were named SNHG5-WT, SNHG5-MUT, BACH1-WT and BACH1-MUT. Then, BC cells were co-transfected with these plasmids and miR-299 mimics or mimics NC. After 48 h transfection, luciferase activity was tested by Dual-Luciferase Reporter Assay System (Promega, WI, USA).

### Western blotting

Total proteins from BC cells were isolated via RIPA buffer (Beyotime, Shanghai, China). After determining the protein concentration with Bradford Protein Assay Kit (Beyotime), equal samples were separated with 10% Sodium Dodecyl Sulfate Polyacrylamide Gel Electrophoresis (SDS-PAGE), follow by transferred onto polyvinylidene difluoride (PVDF) membranes (Millipore, MA, USA). Then membranes were incubated with 5% low-fat milk at room temperature for 3 h. Subsequently, primary antibodies against HK2 (1:1000, Abcam, Cambridge, UK), PFK1 (1:500, Santa Cruz), GAPDH (1:2500, Abcam), β-actin (1:2000, Abcam) were used to probe indicated antigens, and HRP-conjugated secondary antibodies were used to probe primary antibodies. Finally, the protein bands were visualized by electrochemiluminescence (ECL) reagent (Millipore).

### Statistical analysis

All experiments were performed three independent biological replicates in triplicate. Data were presented as mean ± standard deviation (SD) and processed in Graphpad Prism (Version 7.0, USA). Difference between two groups was compared using Student’s t test. One-way analysis of variance (ANOVA) was used to compare the differences among three or more groups. The P < 0.05 was considered to indicate a statistically significant.

## Results

### SNHG5 and BACH1 were upregulated while miR-299 was downregulated in BC cells

**Fig. 1:**
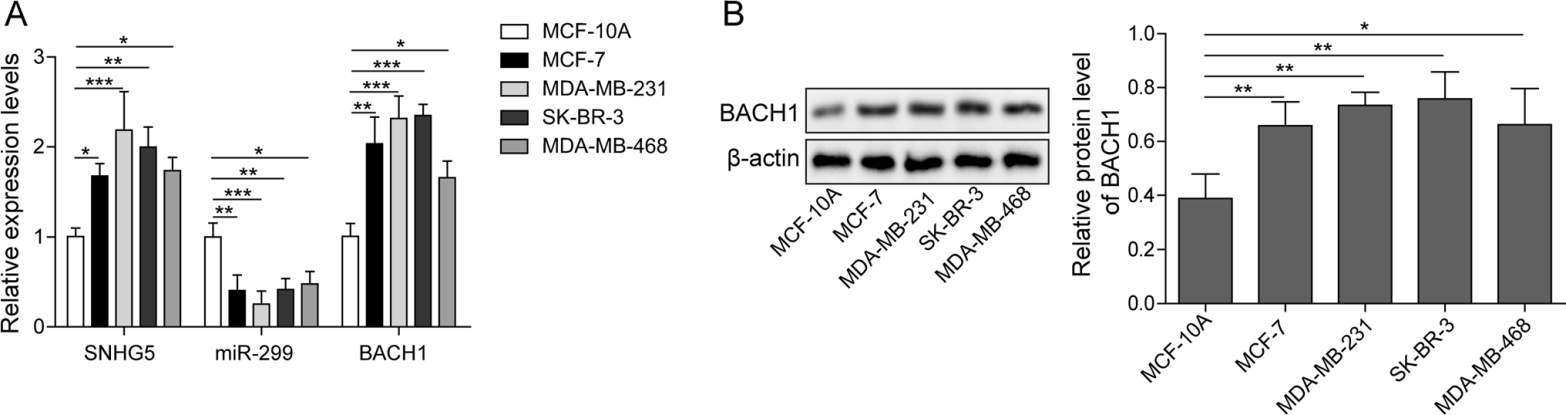
SNHG5 and BACH1 were up-regulated while miR-299 was down-regulated in BC cells. **A** QRT-PCR analysis of SNHG5, miR-299 and BACH1 mRNA expression in BC cell lines. **B** Western blotting detection of BACH1 protein level in BC cell lines. β-Actin was applied as internal control. *p < 0.05, **p < 0.01, ***p < 0.001

We explored the expressions of SNHG5, miR-299 and BACH1 in BC cells (MCF-7, MDA-MB-231, SK-BR-3 and MDA-MB-468), MCF-10A was considered as control. The results demonstrated that SNHG5 and BACH1 levels were significantly up-regulated while miR-299 was down-regulated in different BC cells (Fig. 1A). Next, we observed that BACH1 protein level in BC cells dramatically increased (Fig. 1B). These data indicated that SNHG5 and BACH1 were high expression in BC cells while miR-299 was low expression. MDA-MB-231 and SK-BR-3 cells with the highest expression of SNHG5 were selected for subsequent experiments.

### Silencing of SNHG5 repressed BC cell proliferation and glycolysis

**Fig. 2:**
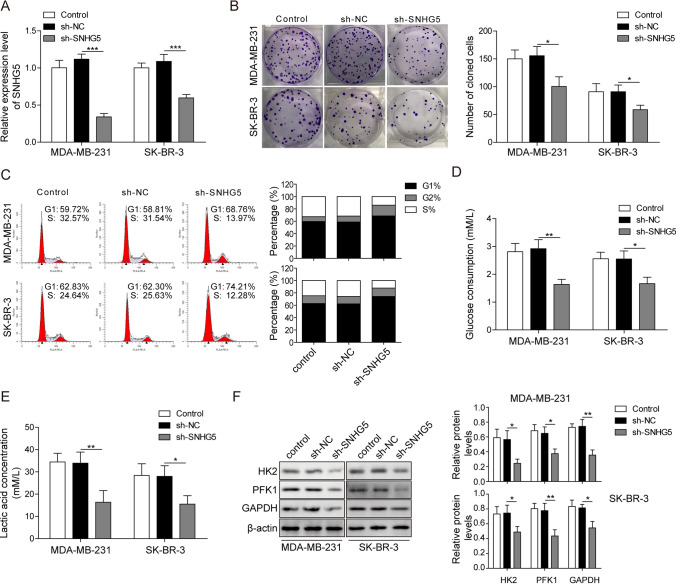
Silencing of SNHG5 repressed BC cell proliferation and glycolysis. **A** QRT-PCR detected the knockdown efficiency of sh-SNHG5. Sh-SNHG5, sh-NC or control were transfected into BC cells. **B** Cell proliferation was analyzed by colony formation assay. **C** Cell cycle was examined by flow cytometry. **D** Glucose consumption and **E** lactate production were analyzed by the corresponding kit. **F** HK2, PFK1 and GAPDH protein expression were determined by western blotting. β-Actin was used as an internal control. *p < 0.05, **p < 0.01

After detecting SNHG5 expression, we analyzed whether SNHG5 silencing had effects on BC development. The results suggested that SNHG5 was markedly decreased by a specific shRNA against SNHG5 (Fig. 2A). As shown in Fig. 2B, knockdown of SNHG5 inhibited cell proliferation. Then the proportion of G1 phase cells in sh-SNHG5 group was increased compared with sh-NC and control groups. This finding indicated that cell cycle was arrested in the G1 phase after silencing of SNHG5 (Fig. 2C). Moreover, the glucose consumption and lactate production of BC cells were dramatically decreased after the knockdown of SNHG5 (Fig. 2D, E). SNHG5 knockdown inhibited the protein expressions of HK2, phosphofructokinase1 (PFK1) and GAPDH in BC cells (Fig. 2F). Taken together, BC cell growth and glycolysis were suppressed by downregulating of SNHG5.

### SNHG5 promoted BACH1 expression by targeting miR-299

**Fig. 3:**
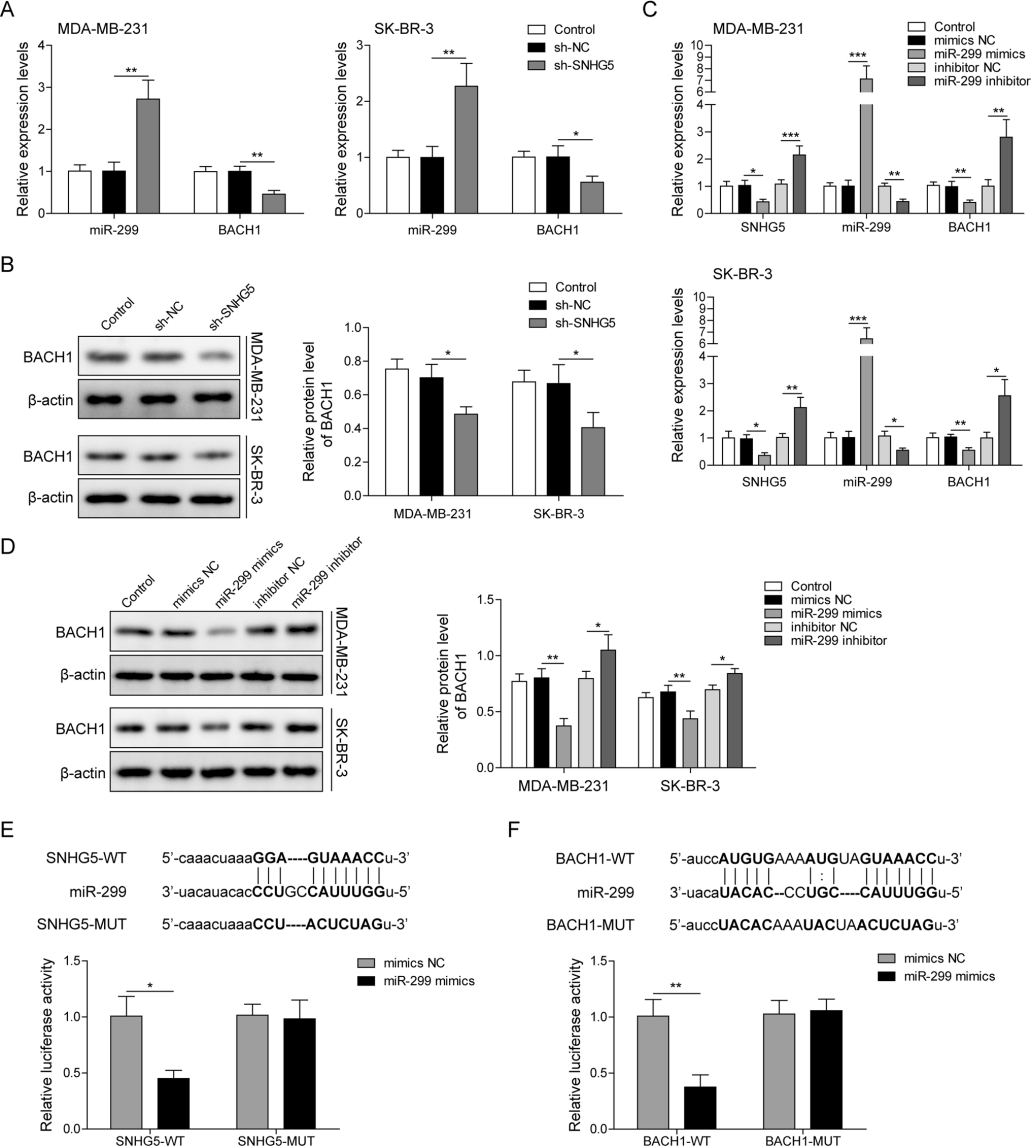
SNHG5 inhibited miR-299 expression while promoted BACH1 expression. **A** qRT-PCR measured miR-299 and BACH1 levels after silencing of SNHG5. **B** BACH1 protein expression was detected by western blotting after knockdown of SNHG5. **C** SNHG5, miR-299 and BACH1 levels were tested by qRT-PCR after transfecting with miR-299 mimics/inhibitor. **D** BACH1 expression was examined after transfecting with miR-299 mimics/inhibitor using western blotting. **E** Upper panel, the binding sites of wild-type or mutant SNHG5 and miR-299. Lower panel, the luciferase activity was analyzed by dual-luciferase reporter assay. **F** Upper panel, potential miR-299 binding sites on the BACH1 3’-UTR. Lower panel, dual-luciferase reporter assay was performed to estimate the luciferase activity. *p < 0.05, **p < 0.01, ***p < 0.001

To further investigate the potential mechanism of SNHG5 in breast cancer development, we firstly detected miR-299 and BACH1 levels after transfecting with sh-SNHG5 in BC cells. Results showed miR-299 was up-regulated while BACH1 was down-regulated in BC cells transfected with sh-SNHG5 (Fig. 3A). And BACH1 protein expression was inhibited in sh-SNHG5 group (Fig. 3B). As shown in Fig. 3C, miR-299 expression was enhanced after transfecting with miR-299 mimics and decreased after transfecting with miR-299 inhibitor. On the contrary, miR-299 overexpression reduced SNHG5 and BACH1 expressions, while inhibition of miR-299 increased their expressions (Fig. 3C). BACH1 protein level was decreased in the miR-299 mimics group and increased in miR-299 inhibitor group (Fig. 3D). Besides, there were binding sites between miR-299 and SNHG5, and the luciferase activity was reduced in SNHG5-WT reporter but not the SNHG5 MUT reporter after co-transfecting with miR-299 and SNHG5-WT or SNHG5-MUT (Fig. 3E). Furthermore, BACH1 might be a target gene of miR-299. After miR-299 and BACH1-WT co-transfection into BC cells, the luciferase activity was repressed while co-transfection of BACH1-MUT reporter showed no evident change (Fig. 3F). These findings suggested that SNHG5 up-regulated BACH1 expression by sponging to miR-299 in BC cells.

### MiR-299 inhibited cell growth and glycolysis in vitro

**Fig. 4:**
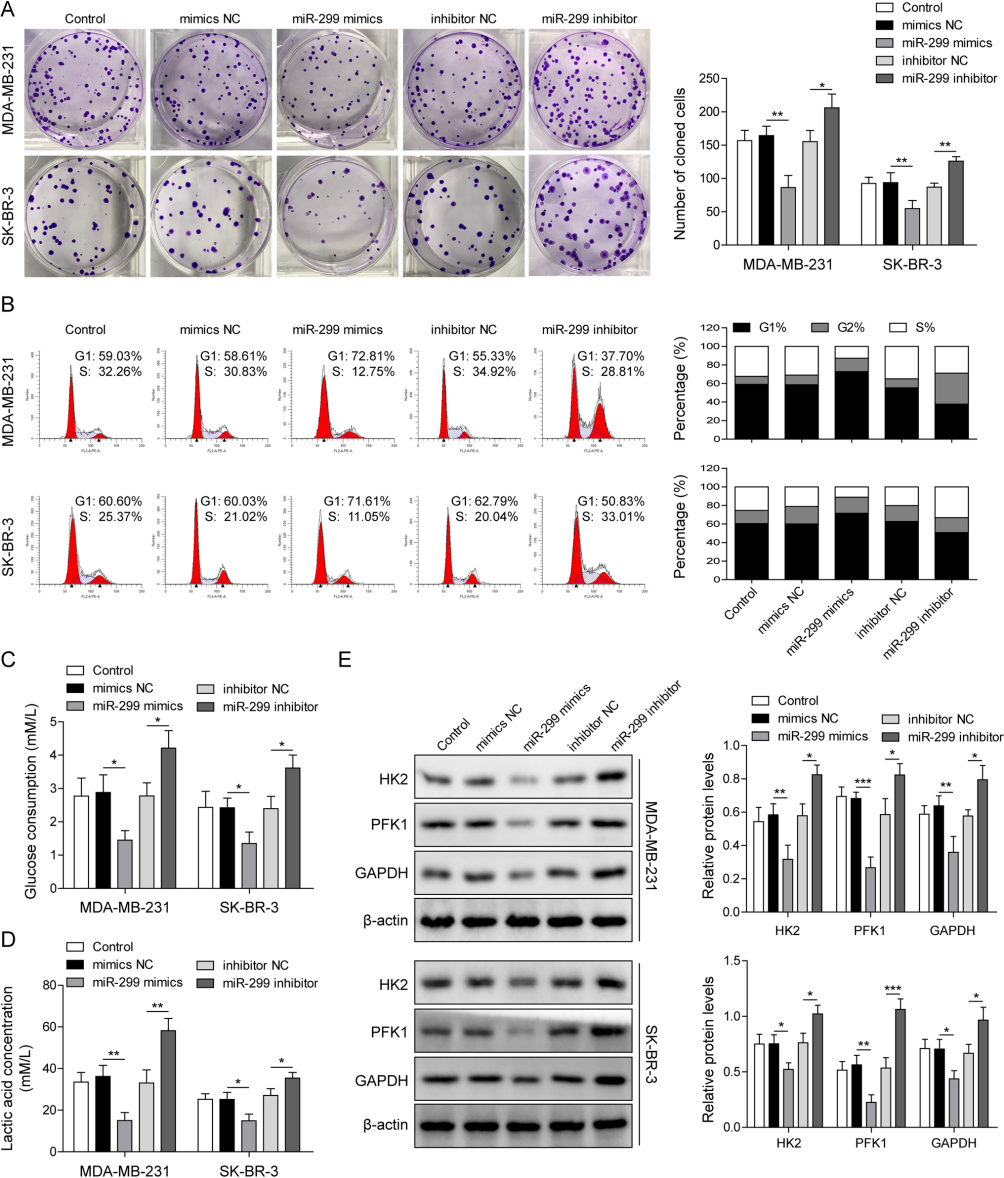
MiR-299 inhibited cell growth and glycolysis in breast cancer. BC cells were transfected with miR-299 mimics and miR-299 inhibitor or their negative control. **A** colony formation assay analyzed cell proliferation. **B** The cell cycle profiles were detected by flow cytometry. **C** Glucose consumption and **D** lactate production were evaluated by kits. **E** The expression of HK2, PFK1 and GAPDH were measured using western blotting. β-Actin was used as an internal control. *p < 0.05, **p < 0.01, ***p < 0.001

To explore whether overexpression or knockdown of miR-299 has effects on BC cell growth, we examined BC cell proliferation after transfecting with miR-299 mimics or inhibitor. The cell proliferation was inhibited in the miR-299 mimics group while the opposite results were observed through silencing of miR-299 (Fig. 4A). Furthermore, the cell cycle was arrested in G1 cell phase after transfecting with miR-299 mimics, while the proportion of S and G2/M phase cells in miR-299 inhibitor group was increased (Fig. 4B). miR-299 overexpression reduced glucose consumption and lactate production, while miR-299 knockdown promoted glycolysis (Fig. 4C, D). Moreover, HK2, PFK1 and GAPDH expressions were inhibited in miR-299 mimics group, while miR-299 silencing promoted their expressions **(**Fig. 4E). Overall, these findings proved that miR-299 inhibited BC cell growth by suppressing cell proliferation and glycolysis.

### MiR-299 inhibition or BACH1 overexpression reversed the inhibition of SNHG5 knockdown on BC cell proliferation and glycolysis

**Fig. 5:**
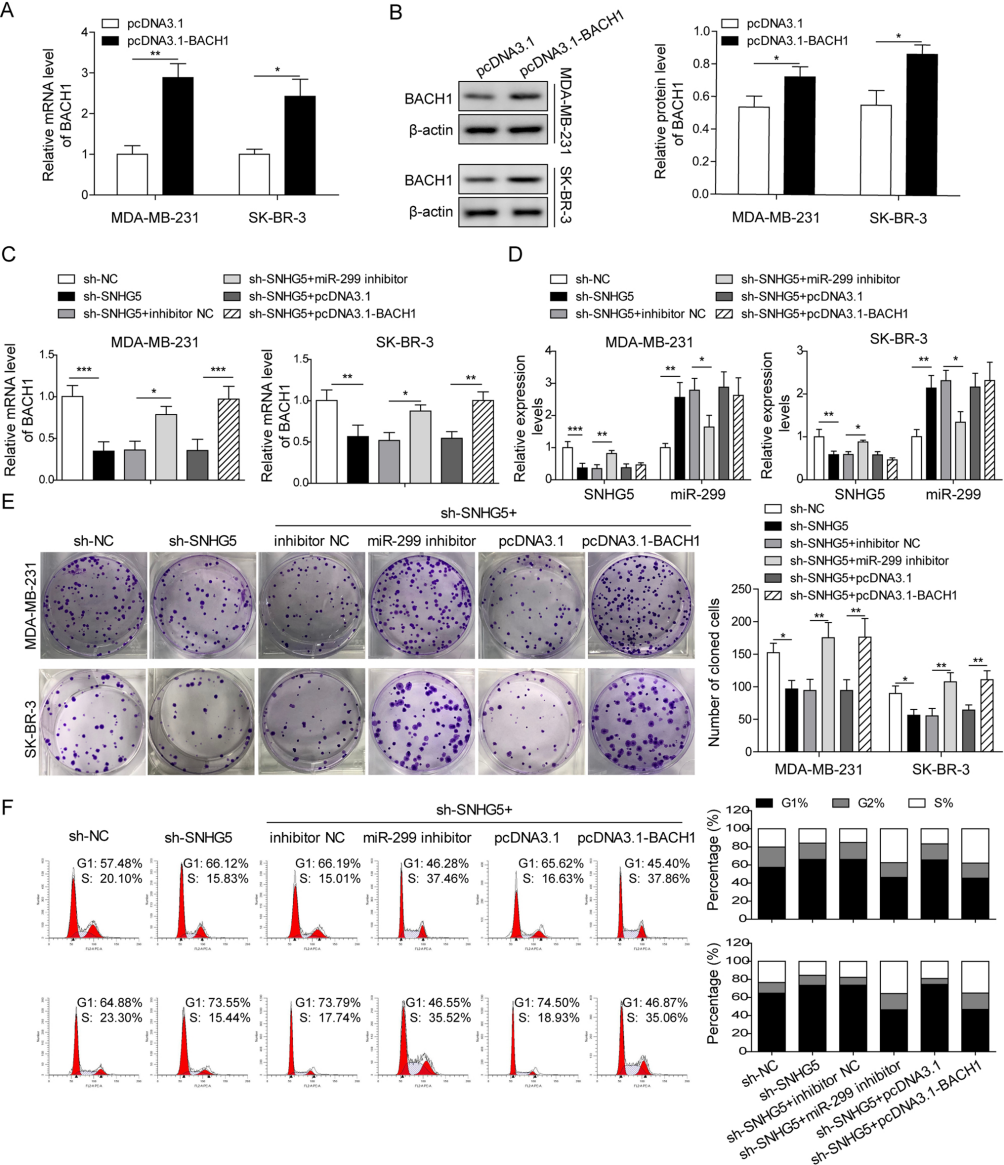
MiR-299 inhibitor or BACH1 overexpression reversed the repressive effects of SNHG5 knockdown on BC cell proliferation and glycolysis. **A** QRT-PCR and **B** western blotting were used to detect the expression of BACH1 in BC cell lines. After transfecting with sh-SNHG5 + miR-299 inhibitor, sh-SNHG5 + pcDNA3.1-BACH1 or their corresponding negative control into BC cells. **C** BACH1 level, **D** SNHG5 and miR-299 expression were detected using qRT-PCR. **E** Colony formation assay, **F** Flow cytometry were used to assess proliferation, cell cycle profiles. *p < 0.05, **p < 0.01, ***p < 0.001

**Fig. 6:**
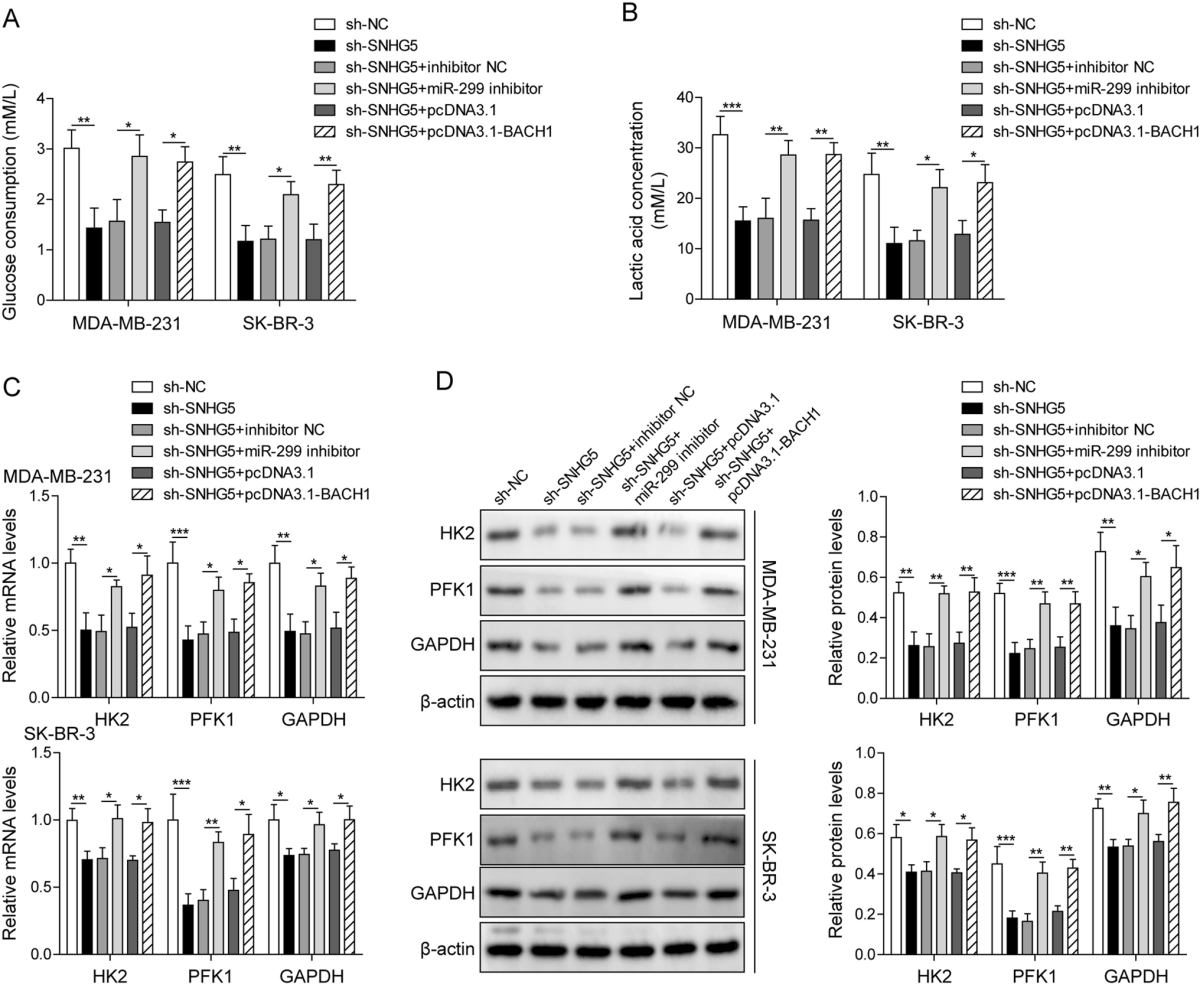
MiR-299 inhibitor or BACH1 overexpression reversed the repressive effects of SNHG5 knockdown on BC cell proliferation and glycolysis. BC cells transfected with sh-SNHG5 + miR-299 inhibitor, sh-SNHG5 + pcDNA3.1-BACH1 or their negative control. **A** Glucose consumption analysis and **B** lactate production were analyzed by kits. **C** HK2, PFK1 and GAPDH mRNA levels, **D** protein levels were detected by qRT-PCR, western blotting. β-Actin was used as an internal control. *p < 0.05, **p < 0.01, ***p < 0.001

To further investigate the role of SNHG5/miR-299/BACH1 axis in BC, we first analyzed the overexpression efficiency of pcDNA3.1-BACH1. The mRNA and protein level of BACH1 was increased after transfecting with pcDNA3.1-BACH1 (Fig. 5A, B). Next, miR-299 inhibitor, pcDNA3.1-BACH1 were transfected into SNHG5 silencing BC cells, the results showed that co-transfection of sh-SNHG5 and miR-299 inhibitor blocked the reduction of BACH1 induced by SNHG5 knockdown. While after co-transfection of sh-SNHG5 and pcDNA3.1-BACH1, SNHG5 and miR-299 levels did not change significantly (Fig. 5C, D). The inhibitory effects of SNHG5 silencing on BC cell proliferation and cell cycle were reversed through miR-299 inhibition or BACH1 overexpression (Fig. 5E, F). Moreover, the glucose consumption and lactate production, as well as HK2, PFK1 and GAPDH levels were also increased in sh-SNHG5 + miR-299 inhibitor and sh-SNHG5 + pcDNA3.1-BACH1 groups **(**Fig. 6A–D**)**, indicating that the inhibitory of sh-SNHG5 on glycolysis were reversed by miR-299 knockdown or BACH1 overexpression. Taken together, SNHG5 promoted the glycolysis through mediating BACH1 by targeting miR-299, thereby promoting BC cell growth.

## Discussion

**Fig. 7:**
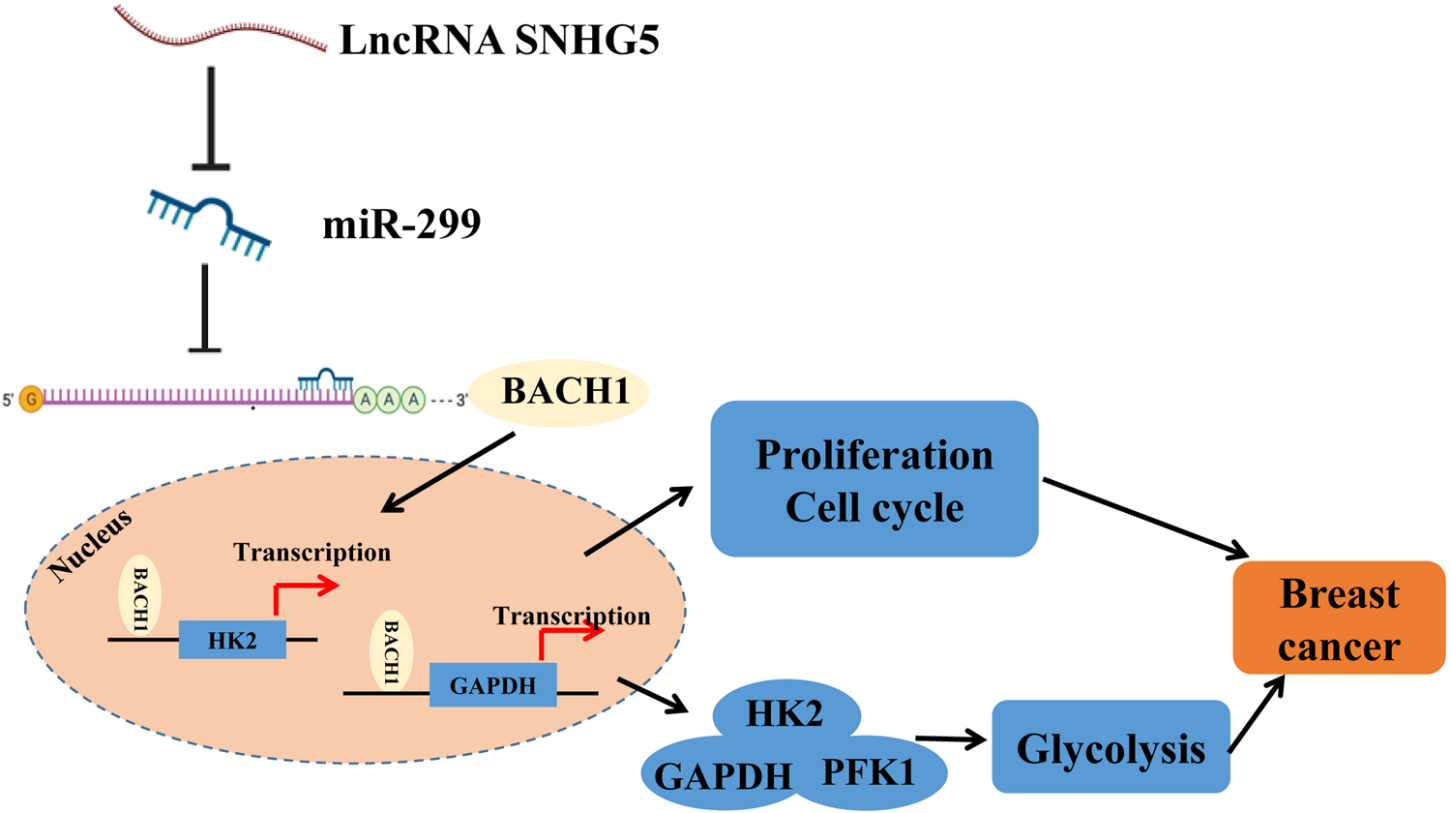
The schematic model diagram of this study. SNHG5 promoted the expression of transcription factor BACH1 by targeting miR-299, and upregalation of BACH1 increased the transcription levels of HK2, PFK1 and GAPDH, thereby promoting cell proliferation, cell cycle and glycolysis in breast cancer

In the worldwide, BC remains a huge public health problem. According to a previous report, 2.1 million BC cases were diagnosed in 2018, accounting for almost a quarter of all cancer cases among women [22]. Glycolysis acts as an essential glucose metabolism process that converts glucose to pyruvate, ultimately producing lactic acid, which plays an important role in the maintenance of tumor cells [23]. Since tumor cells rely on glycolysis to obtain growth energy, increased glycolysis is already a prominent feature of several cancers, including BC, and has been successfully applied to cancer diagnosis [24]. In the present study, we first found that knockdown of SNHG5 could inhibit cell proliferation and glycolysis in BC cells through regulating BACH1 expression via miR-299 (Fig. 7).

Previous reports proved that lncRNAs, such as H19 [25], were associated with poor overall survival in a different type of cancers by promoting glycolysis. Furthermore, increasing evidence indicated that SNHG5 played important regulatory roles in proliferation, migration, invasion and growth of various tumor cells [26, 27]. In the study of BC, Chi et al. indicated that SNHG5 up-regulated proliferating cell nuclear antigen (PCNA) to promote proliferation and cell cycle progression by targeting miR-154-5p [19]. Here, we also found SNHG5 was highly expressed in BC cells. Through functional experiments, we observed that SNHG5 knockdown suppressed BC cell proliferation and cell cycle progression. In addition, a study showed Nur77-activated WFDC21P significantly inhibited the proliferation and metastasis of hepatocellular carcinoma cells by inhibiting glucose uptake and lactate production [28]. LncRNA FGF13-AS1 suppressed proliferation, migration and invasion of BC cells by inhibiting glycolysis and stem cell properties [29]. However, the research of SNHG5 in glycolysis was rarely investigated. We revealed that SNHG5 silencing impaired the glucose consumption and lactate production of BC cells, and suppressed HK2, PFK1 and GAPDH levels, which are indispensable regulatory enzymes of glycolysis [28].

Previous studies have shown that lncRNAs function as competitive endogenous RNAs (ceRNAs) by sponging miRNA to negatively regulate the expression of miRNAs and their downstream target genes. In our study, dual-luciferase assay results demonstrated that the luciferase activity was inhibited in BC cells co-transfected with miR-299 mimics and SNHG5-WT. Moreover, transfection with miR-299 inhibitor reversed the inhibitory effect of sh-SNHG5 on BC cell proliferation and glycolysis. Accumulating studies demonstrated miR-299 was downregulated and inhibited tumor growth in various cancers, such as nasopharyngeal carcinoma [30] and thyroid cancer [31]. Furthermore, a study proved that compared to other miRNAs expression, miR-299 was the lowest expressed in metastatic BC, while the expression returned to normal after treatment [32]. Li et al. observed that miR-299 acted as a tumor suppressor to inhibit BC cell migration and invasion by targeting STK39 [21]. Inhibition of miR-125a promoted endothelial cell tube formation by increasing PKM2 expression [33]. miR-186 effectively inhibited the tamoxifen resistance of BC cells by inhibiting the glycolysis in vitro [34]. In this study, we found that miR-299 inhibited cell proliferation, cell cycle and glycolysis in BC. Taken together, miR-299 acted as a tumor suppressor in BC, possibly by inhibiting the glycolysis.

According to a previous study, BACH1 has shown complex function in cancer [35]. Numerous studies showed that BACH1 promoted BC cell invasion and metastasis [36, 37]. Furthermore, Lee et al. suggested that BACH1 regulated pyruvate dehydrogenase kinase (PDK) transcription and BACH1 silencing decreased glycolysis progression in BC cells [9]. Our findings supported these observations. MiR-142-3p reduced cell proliferation, invasion and migration of human breast cancer by regulating BACH1 expression [38]. A previous study showed that BACH1, as a transcription factor, promoted transcription of HK2 and GAPDH by binding to their promoters [15]. In our study, BACH1 overexpression promoted cell proliferation, cell cycle, glucose consumption and lactate production in BC, as well as reversed the inhibitory effect of SNHG5 silencing on BC cells. Besides, SNHG5 knockdown decreased BACH1 expression, while the inhibition effect was reserved by co-transfecting miR-299 inhibitor, suggesting that SNHG5 regulated BACH1 by targeting miR-299. Taken together, our data, for the first time, indicated that BACH1 acted as a direct target of miR-299, which was negatively regulated by miR-299 in BC cells, and we have also proved a new regulatory pathway of glycolysis in BC cells, namely SNHG5/miR-299/BACH1 axis.

In summary, our study provided evidence that knockdown of SNHG5 suppressed the growth of BC cells, and reduced glycometabolism-related proteins levels, meanwhile inhibited the glucose consumption and lactate secretion, suggesting SNHG5 activated BACH1 expression to promote BC cell growth and glycolysis by targeting miR-299. It might improve the diagnostic and therapeutic approaches of BC.

## Data Availability

All data generated or analyzed during this study are included in this article. The datasets used and/or analyzed during the current study are available from the corresponding author on reasonable request.
